# Sliding-Scale versus Basal-Bolus Insulin in the Management of Severe or Acute Hyperglycemia in Type 2 Diabetes Patients: A Retrospective Study

**DOI:** 10.1371/journal.pone.0106505

**Published:** 2014-09-02

**Authors:** Hasniza Zaman Huri, Vishaaliny Permalu, Shireene Ratna Vethakkan

**Affiliations:** 1 Department of Pharmacy, Faculty of Medicine, University of Malaya, Kuala Lumpur, Malaysia; 2 Clinical Investigation Centre, University Malaya Medical Centre, Lembah Pantai, Kuala Lumpur, Malaysia; 3 Endocrinology Unit, Department of Medicine, Faculty of Medicine, University of Malaya, Kuala Lumpur, Malaysia; University of Michigan Medical School, United States of America

## Abstract

Sliding-scale and basal-bolus insulin regimens are two options available for the treatment of severe or acute hyperglycemia in type 2 diabetes mellitus patients. Although its use is not recommended, sliding-scale insulin therapy is still being used widely. The aims of the study were to compare the glycemic control achieved by using sliding-scale or basal-bolus regimens for the management of severe or acute hyperglycemia in patients with type 2 diabetes and to analyze factors associated with the types of insulin therapy used in the management of severe or acute hyperglycemia. This retrospective study was conducted using the medical records of patients with acute or severe hyperglycemia admitted to a hospital in Malaysia from January 2008 to December 2012. A total of 202 patients and 247 admissions were included. Patients treated with the basal-bolus insulin regimen attained lower fasting blood glucose (10.8±2.3 versus 11.6±3.5 mmol/L; p = 0.028) and mean glucose levels throughout severe/acute hyperglycemia (12.3±1.9 versus 12.8±2.2; p = 0.021) compared with sliding-scale insulin regimens. Diabetic ketoacidosis (p = 0.043), cardiovascular diseases (p = 0.005), acute exacerbation of bronchial asthma (p = 0.010), and the use of corticosteroids (p = 0.037) and loop diuretics (p = 0.016) were significantly associated with the type of insulin regimen used. In conclusion, type 2 diabetes patients with severe and acute hyperglycemia achieved better glycemic control with the basal-bolus regimen than with sliding-scale insulin, and factors associated with the insulin regimen used could be identified.

## Introduction

Diabetes mellitus is a significant global health disorder. Type 2 diabetes mellitus (T2DM) is becoming more common in almost every population, accounting for approximately 90% of all cases of diabetes in adults in Malaysia in 2008 [1]. Severe or acute hyperglycemia is an acute manifestation of diabetes that commonly occurs in T2DM patients, and requires intensive treatment and hospitalization [2]. According to a prospective cohort study, the causes of admission to hospital in T2DM patients with hyperglycemia include diabetic ketoacidosis (DKA), hyperosmolar hyperglycemic state and serious infections [3]. In addition, the concurrent use of blood-glucose altering medications such as corticosteroids, antipsychotics and diuretics tend to worsen severe or acute hyperglycemia by increasing hepatic gluconeogenesis as well as impairing peripheral glucose uptake [2].

Despite the available treatment options for severe or acute hyperglycemia in T2DM patients, glycemic control in this population remains suboptimal [4]. This is partly attributable to the continued use of sliding-scale insulin regimens to manage severe or acute hyperglycemia, despite many treatment guidelines [5], recommending against its use. In addition, there are limited local and global data on the level of glycemic control achieved in T2DM patients with severe or acute hyperglycemia based on the type of insulin regimen used. Therefore, this study was conducted to identify the treatment approach and the achievement of glycemic control among hospitalized T2DM patients with severe or acute hyperglycemia. The specific objectives were twofold: (i) to compare the glycemic control achieved by using sliding-scale (Actrapid or basal-bolus (Actrapid and Insulatard) regimens for the management of severe or acute hyperglycemia in T2DM patients; and (ii) to analyze factors associated with the types of insulin therapy used in the management of severe or acute hyperglycemia.

## Methodology

### Study Population

**Figure 1:**
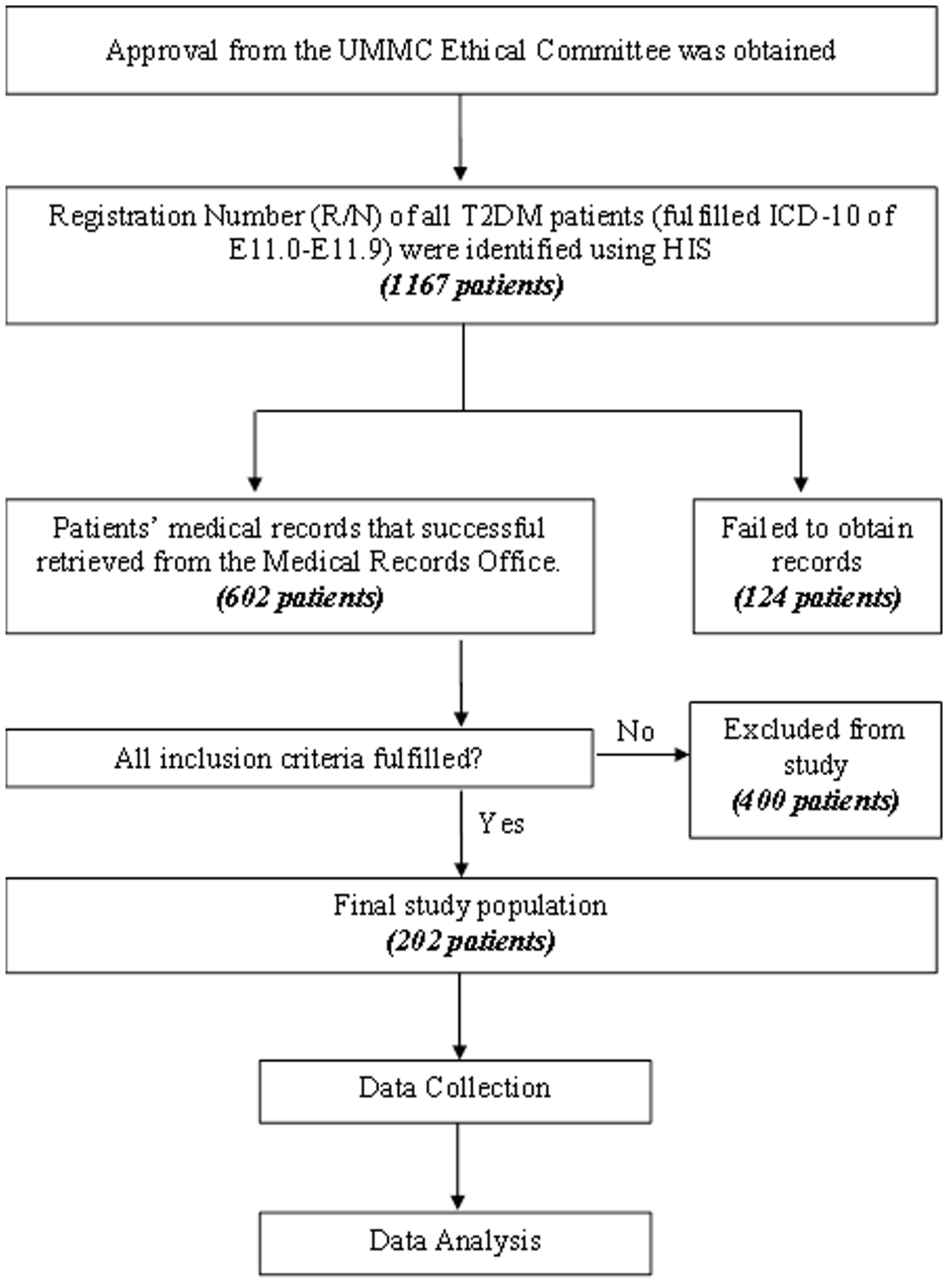
Flow chart of methodology. ICD-10 =  International Statistical Classification of Diseases and Related Problems 10^th^ Revision; UMMC  =  University of Malaya Medical Clinic.

This retrospective study consisted of T2DM patients with severe or acute hyperglycemia admitted to the University of Malaya Medical Centre (UMMC), a principal 1000-bed teaching hospital in Kuala Lumpur, Malaysia, from January 2008 to December 2012. The study was conducted in accordance with the Declaration of Helsinki and was approved by the medical ethics committee of the UMMC (reference number 956.32). The committee waived the need for written informed consent from participants. The registration numbers of 1167 patients with T2DM according to International Statistical Classification of Diseases and Related Health Problems 10^th^ Revision (ICD-10) codes E11.0–E11.9 were identified via the Hospital Information System. Of these 1167 patients, medical records for 602 patients were successfully traced. Using the convenient sampling method, 202 patients who fulfilled the inclusion criteria (see below) were included in this study. An overview of the study methodology is shown in Figure 1.

### Inclusion Criteria

1. Adult T2DM patients who are equal or more than 18 years old
2. Hospitalized with severe or acute hyperglycemia with blood glucose level over 13.9 mmol/L
3. Admitted to general medical units
4. Treated only with insulin during the hospitalization

### Exclusion Criteria

1. Patients with other types of diabetes mellitus
2. Patient with incomplete data

### Data Collection

The following patient data were collected:

- demographic characteristics (age, sex, ethnicity, and body mass index [BMI]).
- comorbidities.
- the use of concurrent medications;
- blood glucose levels on admission and throughout the severe or acute hyperglycemia phase; and
- laboratory results and other monitoring parameters as stated in case notes.

### Assessment of Glycemic Control

Patients were monitored to evaluate glycemic control throughout the severe or acute hyperglycemia phase. Assessment of glycemic control was based on the glucose readings measured during treatment. Glycemic targets were defined according to American Diabetes Association (ADA) recommendations (American Diabetes Association, 2013), i.e., fasting plasma glucose <7.0 mmol/L; pre-meal plasma glucose and overall blood glucose <10 mmol/L.

### Statistical Techniques

Collected data were pooled and analyzed using IBM SPSS Statistics Version 20.0 (Armonk, New York, USA). Kolmogorov-Smirnov was used to test for normality of continuous data. Normally distributed data was expressed as mean ± standard deviation whereas data that was not normally distributed was expressed as median (interquartile range). Continuous data were expressed as mean ±standard deviation while categorical data were expressed as percentages. The association between categorical variables was examined using the Pearson Chi Square test with Continuity correction and Fisher's exact adjustment when necessary. The t-test was used to evaluate the differences in means between groups of continuous data. Significance was set at p<0.05. The minimum sample size was calculated using Epi Info™ Program Version 7.0 (Centers for Disease Control and Prevention, Atlanta, USA). A minimum of 108 patients were needed to detect a minimum difference of 1 mmol/L, a power of β = 0.8 and a confidence level of 95%.

## Results

### Demographic Characteristics

A total of 202 T2DM patients with severe or acute hyperglycemia on admission were included in this study of a total of 247 hospital admissions. There were slightly more female than male patients, and the most common ethnicity was Malay (42.6%), followed by Indians (38.6%), Chinese (17.3%) and others (1.5%).

A total of 73.8% and 26.2% of the study population was non-elderly (≥18 years) and elderly (≥65 years), respectively. Data were available on BMI for 28.7% of patients, 12.9% of whom had BMI in the normal range, followed by pre-obese (7.9%), obese (5.9%) and underweight (2%) (see Table 1).

**Table 1:** Demographic characteristics of the patients (N = 202).

Demographic characteristics	Number of patients (Percentage, %)
**Gender**	
Male	96 (47.5)
Female	106 (52.5)
**Age**	
Non-elderly (≥18 years)	149 (73.8)
Elderly (≥65 years)	53 (26.2)
**Ethnicity**	
Malay	86 (42.6)
Indian	78 (38.6)
Chinese	35 (17.3)
Others	3 (1.5)
**BMI (kg/m^2^)** a	
Underweight (<18.5)	4 (2.0)
Normal range (18.5–24.9)	26 (12.9)
Pre-obese (25–29.9)	16 (7.9)
Obese (≥30)	12 (5.9)
Unknown	144 (71.3)

a Based on data available for 28.7% of patients.

BMI  =  body mass index.

### Clinical Characteristics

Clinical characteristics of the patients are shown in Table 2. Of the 202 patients, more than 50% of the patients were hospitalized for ≤7 days, with a minimum stay of 2 days. The mean duration of the 247 admissions was 7.9±6.3 days.

**Table 2:** Clinical characteristics of the patients (N = 202).

Clinical characteristics	N	Number of patients (Percentage, %)
**Duration of hospital stay**	202	
Not more than 7 days		125 (61.9)
8 to 14 days		50 (24.8)
More than 15 days		27 (13.4)
**Admission blood glucose levels (mmol/L)**	202	
12.3–14.4		5 (2.5)
14.5–16.7		27 (13.4)
16.8–19.4		35 (17.3)
19.5–22.2		37 (18.3)
>22.3		98 (48.5)
**HbA_1c_**	202	
Achieve target (<6.5)		0 (0)
Not achieve target (≥6.5		92 (45.5)
Unknown		110 (54.5)
↑ **Hyperglycemia occurred secondary to or was caused by**	247	
Infection		111 (44.9)
Diabetic ketoacidosis		33 (13.4)
Uncontrolled diabetes (non-compliance)		33 (13.4)
Cardiovascular disease		32 (13.0)
Cerebrovascular accident		9 (3.6)
Renal impairment		8 (3.2)
Hyperosmolar hyperglycemia		7 (2.8)
Acute exacerbation of bronchial asthma		6 (2.4)
Others		8 (3.2)
* **Comorbidities**	202	
Hypertension		125 (61.9)
Ischemic heart disease		38 (18.8)
Renal Impairment		34 (16.8)
Dyslipidemia		19 (9.4)
Stroke		12 (5.9)
Bronchial Asthma		9 (4.5)
Liver Impairment		3 (1.5)
Infection		2 (1.0)
Pneumonia		1 (0.5)
Others		8 (4.0)

↑ One patient may have more than one hospital admission and been treated with both basal-bolus insulin and sliding-scale insulin;

* One patient may have more than one comorbidity.

Blood glucose levels on admission were normally distributed with a mean of 24.4±9.3 mmol/L. Almost half of the patients (48.5%) were admitted to hospital with a blood glucose level >22.3 mmol/L, with a maximum of 65.3 mmol/L. Overall, mean hemoglobin (Hb) A_1c_ was 11.7%±2.6% (104 mmol/mol ±28.4 mmol/mol).

The most common cause of severe or acute hyperglycemia among the admitted patients was infection, accounting for 44.9% of admissions, followed by DKA (13.4%), uncontrolled diabetes secondary to non-compliance (13.4%), and cardiovascular disease (13%). The majority of patients (72.5%) had more than one comorbidity; only 27.5% of patients had no comorbidities. Hypertension was the most frequent comorbidity, reported in 61.9% of patients, followed by ischemic heart disease (18.8%) and renal impairment (16.8%).

### Insulin Regimens Used during Severe or Acute Hyperglycemia

Admissions were evaluated based on the insulin regimen used to manage the severe or acute hyperglycemia. A total of 338 cases were evaluated for insulin use. Sliding-scale insulin and basal-bolus insulin was used in 53% and 47% of admissions, respectively.

### Glycemic Control achieved with Insulin Regimens

Table 3 shows the level of glycemic control achieved with each regimen. Of the 338 cases, 159 were treated using basal-bolus insulin, and 179 cases were treated using sliding-scale insulin. Patients treated with the basal-bolus insulin regimen attained lower fasting blood glucose (10.8±2.3 versus 11.6±3.5 mmol/L; p = 0.028) and mean glucose levels (12.3±1.9 versus 12.8±2.2; p = 0.021) throughout severe or acute hyperglycemia compared with sliding-scale insulin regimens.

**Table 3:** Level of glycemic control achieved with the two insulin regimens.

	Basal-bolus Insulin	Sliding-scale insulin	p-value
**Number of cases** ↑	159	179	
**Number of blood glucose readings**	2766	4015	
**Mean fasting blood glucose (mmol/L)**	10.8±2.3	11.6±3.5	0.028*
**Mean blood glucose throughout severe or acute hyperglycemia (mmol/L)**	12.3±1.9	12.8±2.2	0.021*
**Mean insulin doses**	12.51±5.5 units	3.14±0.9 units/h	
**Blood glucose <3.3 mmol/L**			
Number of cases (%)	4 (2.5)	18 (10.1)	0.005*
Number of readings (%)	5 (0.18)	21 (0.52)	
**Blood glucose <3.9 mmol/L**			
Number of cases (%)	9 (5.7)	17 (9.5)	0.186
Number of readings (%)	14 (0.51)	18 (0.45)	

↑ One patient may have more than one hospital admission and been treated with both basal-bolus insulin and sliding-scale insulin;

* Statistically significant (p<0.05).

### Factors Associated with the Management of Severe or Acute Hyperglycemia

#### Causes of Severe or Acute Hyperglycemia

DKA, cardiovascular diseases and acute exacerbation of bronchial asthma were significantly associated with the insulin regimen used (Table 4). The use of sliding-scale insulin (67.3%) was more common than that of basal-bolus insulin (32.7%) among patients with DKA. In contrast, when compared with sliding-scale insulin, basal-bolus insulin was more frequently used in managing severe or acute hyperglycemia secondary to cardiovascular diseases and acute exacerbation of bronchial asthma (15 and 6 cases, respectively).

**Table 4:** Causes of severe or acute hyperglycemia based on the insulin regimens.

Causes of severe or acute hyperglycemia		Insulin regimens		p-value
		Basal-bolus (n = 159)	Sliding-scale (n = 179)	
**Diabetic ketoacidosis**	Present	16 (32.7%)	33 (67.3%)	0.043*
	Not Present	143 (49.5%)	146 (50.5%)	
**Cardiovascular diseases**	Present	27 (69.2%)	12 (30.8%)	0.005*
	Not Present	132 (44.1%)	167 (55.9%)	
**Acute exacerbation of bronchial asthma**	Present	6 (100%)	0 (0%)	0.010*
	Not Present	153 (46.1%)	179 (53.9%)	

* Statistically significant (p<0.05).

### Concomitant Drug Use during Severe or Acute Hyperglycemia

Regarding concomitant drug use, corticosteroids (p = 0.037), and loop diuretics (p = 0.016) appeared to be significantly associated with basal-bolus and sliding-scale insulin regimens (Table 5).

**Table 5:** Comparison of drug use based on the insulin regimen used.

Drug Class		Insulin regimens		p-value
		Basal-bolus (n = 159)	Sliding-scale (n = 179)	
**Corticosteroids**	Present	15 (71.4%)	6 (28.6%)	0.037*
	Not Present	144 (45.4%)	173 (54.6%)	
**Loop diuretics**	Present	22 (68.8%)	10 (31.3%)	0.016*
	Not Present	137 (44.8%)	169 (55.2%)	

*Statistically significant (p<0.05).

**Figure 2:**
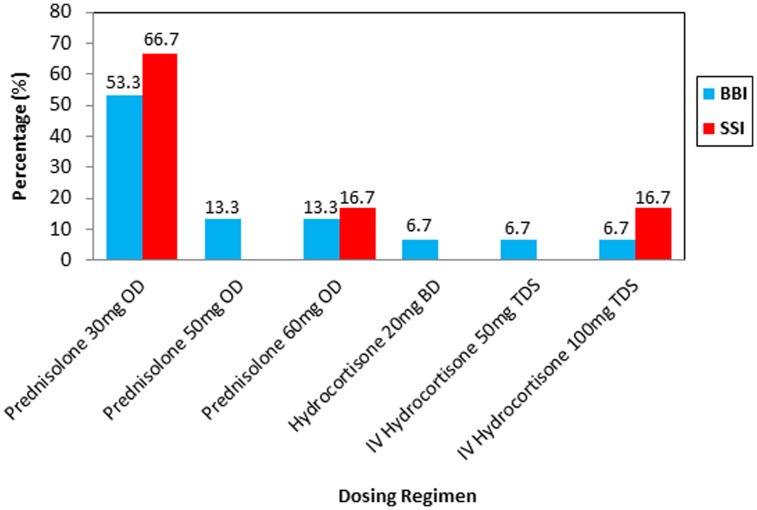
Corticosteroid dosing regimens used. BBI  =  basal-bolus insulin, SSI  =  sliding-scale insulin, OD  =  once daily, BD  =  twice daily, TDS  =  three times daily, IV  =  intravenous.

Figure 2 shows the common dosing regimens of corticosteroids administered throughout the severe or acute hyperglycemia phase stratified by insulin regimen (15 cases using basal-bolus insulin and six cases using sliding-scale insulin). Oral administration of prednisolone 30 mg was the most common corticosteroid dosing regimen among the sliding-scale insulin and basal-bolus insulin-treated cases, comprising 66.7% and 53.3% of cases, respectively.

Factors not associated with the Management of Severe or Acute Hyperglycemia

Factors that had no significant association with the management of severe or acute hyperglycemia are shown in Tables 6–8.

**Table 6:** Comparison of demographic and clinical characteristics based on insulin regimens.

		Insulin regimens^a^		p-value
		Basal-bolus (n = 159)	Sliding-scale (n = 179)	
**Demographic characteristics**				
**Gender**	Male	75 (47.2%)	82 (45.8%)	0.802
	Female	84 (52.8%)	97 (54.2%)	
**Age**	Non-elderly	116 (73%)	134 (74.9%)	0.690
	Elderly	43 (27%)	45 (25.1%)	
**Causes of severe or acute hyperglycemia**				
**Cerebrovascular accident**	Present	5 (3.1%)	5 (2.8%)	1.000
	Not present	154 (96.9%)	174 (97.2%)	
**Uncontrolled diabetes**	Present	19 (11.9%)	28 (15.6%)	0.411
	Not present	140 (88.1%)	151 (84.4%)	
**Renal impairment**	Present	5 (3.1%)	6 (3.4%)	1.000
	Not present	154 (96.9%)	173 (96.6%)	
**Others**	Present	6 (3.8%)	4 (2.2%)	0.525^b^
	Not present	153 (96.2%)	175 (97.8%)	
**Comorbidities**				
**Hypertension**	Present	103 (64.8%)	109 (60.9%)	0.532
	Not present	56 (35.2%)	70 (39.1%)	
**Ischemic heart disease**	Present	38 (23.9%)	27 (15.1%)	0.056
	Not present	121 (76.1%)	152 (84.9%)	
**Stroke**	Present	11 (6.9%)	13 (7.3%)	1.000
	Not present	148 (93.1%)	166 (92.7%)	
**Pneumonia**	Present	1 (0.6%)	0 (0%)	0.470^b^
	Not present	158 (99.4%)	179 (100%)	
**Infection**	Present	2 (1.3%)	2 (1.1%)	1.000^b^
	Not present	157 (98.7%)	177 (98.9%)	
**Dyslipidemia**	Present	15 (9.4%)	13 (7.3%)	0.599
	Not present	144 (90.6%)	166 (92.7%)	
**Renal Impairment**	Present	27 (17%)	27 (15.1%)	0.744
	Not present	132 (83%)	152 (84.9%)	
**Liver Impairment**	Present	5 (3.1%)	2 (0.6%)	0.260^b^
	Not present	154 (96.9%)	177 (98.9%)	
**Bronchial Asthma**	Present	9 (5.7%)	8 (4.5%)	0.802
	Not present	150 (94.3%)	171 (95.5%)	
**Others**	Present	6 (3.8%)	8 (4.5%)	0.963
	Not present	153 (96.2%)	171 (95.5%)	

a One patient may have more than one hospital admission and been treated with both basal-bolus insulin and sliding-scale insulin.

b Computed using Fisher's Exact Test.

**Table 7:** Comparison of drug use according to insulin regimen used.

		Insulin regimens^a^		p-value
		Basal-bolus (n = 159)	Sliding-scale (n = 179)	
**Beta blockers**	Present	23 (14.5%)	19 (10.6%)	0.365
	Not present	136 (85.5%)	160 (89.4%)	
**Calcium channel blockers**	Present	37 (23.3%)	29 (16.2%)	0.134
	Not present	122 (76.7%)	150 (83.8%)	
**Alpha-1 adrenergic blockers**	Present	6 (3.8%)	2 (1.1%)	0.155^b^
	Not present	153 (96.2%)	177 (98.9%)	
**Angiotensin-converting enzyme inhibitors**	Present	32 (20.1%)	22 (12.3%)	0.070
	Not present	127 (79.9%)	157 (87.7%)	
**Cephalosporins**	Present	38 (23.9%)	48 (26.8%)	0.625
	Not present	121 (76.1%)	131 (73.2%)	
**Penicillins**	Present	63 (39.6%)	74 (41.3%)	0.834
	Not present	96 (60.4%)	105 (58.7%)	
**Macrolides**	Present	13 (8.2%)	13 (7.3%)	0.912
	Not present	146 (91.8%)	166 (92.7%)	
**Fluoroquinolones**	Present	0 (0%)	1 (0.6%)	1.000^b^
	Not present	159 (100%)	178 (99.4%)	
**Opiod analgesics**	Present	18 (11.32%)	19 (10.6%)	0.974
	Not present	141 (88.7%)	160 (89.4%)	
**Atypical antipsychotics**	Present	1 (0.6%)	1 (0.6%)	1.000^b^
	Not present	158 (99.4%)	178 (99.4%)	
**Proton-pump inhibitors**	Present	14 (8.8%)	11 (6.1%)	0.469
	Not present	145 (91.2%)	168 (93.9%)	
**Histamine-2 receptor antagonist**	Present	23 (14.5%)	29 (16.2%)	0.771
	Not present	136 (85.5%)	150 (83.8%)	

a One patient may have more than one hospital admission and been treated with both basal-bolus insulin and sliding-scale insulin.

b Computed using Fisher's Exact Test.

**Table 8:** Comparison of corticosteroid dosing regimen used according to insulin regimen used.

Corticosteroids		Insulin regimens		p-value
		Basal-bolus (n = 15)	Sliding-scale (n = 6)	
**PO Prednisolone 30 mg OD**	Present	8 (53.3%)	4 (66.7%)	0.659^a^
	Not present	7 (46.7%)	2 (33.3%)	
**PO Prednisolone 50 mg OD**	Present	2 (13.3%)	0 (0%)	1.000^a^
	Not present	13 (86.7%)	6 (100%)	
**PO Prednisolone 60 mg OD**	Present	2 (13.3%)	1 (16.7%)	1.000^a^
	Not present	13 (86.7%)	5 (83.3%)	
**PO Hydrocortisone 20 mg BD**	Present	1 (6.67%)	0 (0%)	1.000^a^
	Not present	14 (93.3%)	6 (100%)	
**IV Hydrocortisone 50 mg TDS**	Present	1 (6.67%)	0 (0%)	1.000^a^
	Not present	14 (93.3%)	6 (100%)	
**IV Hydrocortisone 100 mg TDS**	Present	1 (6.67%)	1 (16.7%)	0.500^a^
	Not present	14 (93.3%)	5 (83.3%)	

a Computed using Fisher's Exact Test.

## Discussion

### Demographic Characteristics

Of the 202 patients, majority were female. Malays comprised the highest, followed by the Indian patients. The observed difference in ethnic distribution might be attributable to the stringent inclusion criteria of this study, where only T2DM patients with severe or acute hyperglycemia on admission were considered for analysis.

The proportions of obese and pre-obese patients were higher in a study conducted by Zaman Huri *et al*. [3], in which 46.2% and 37.2% of the patients were obese and pre-obese, respectively. However, this is in contrast to the current study where, in the 28.7% of the study population for whom data were available, 12.9% of the study population had a BMI in the normal range, and 7.9% and 5.9% were classified as pre-obese and obese, respectively.

### Clinical Characteristics

The mean duration of the 247 hospital admissions was 7.9 days, similar to that in a retrospective study involving T2DM patients in which 71.5% of patients stayed in hospital for not more than seven days and 9.5% were hospitalized for more than 15 days [3].

Data on HbA_1c_ were available for 45.5% of patients in our study. The mean HbA_1c_ value was 11.7% (104 mmol/mol). However, a mean HbA_1c_ of 7.7% (61 mmol/mol) was reported in a study by Umpierrez *et al*. [6]. The higher HbA_1c_ in the current study reflects the poor glycemic control among study subjects and is presumably associated with the development of severe or acute hyperglycemia leading to hospitalization.

The most cause of severe or acute hyperglycemia in this study was infection, comprising 44.9% of admissions. Other reasons for admission included DKA, uncontrolled diabetes secondary to non-compliance, and cardiovascular diseases. Cardiovascular diseases and non-compliance to diabetes medications were found to be common among the 156 patients, comprising 7.1% and 8.3% of cases, respectively.

This study also revealed that 72.5% of the studied patients had more than one comorbidity, with hypertension being the most common (61.9%), followed by ischemic heart disease (18.8%) and renal impairment (16.8%). Zaman Huri *et al*. [3] reported a similar pattern, where hypertension was the most common comorbidity (82.7%), followed by renal impairment (39.7%) and ischemic heart disease (27.5%). Insulin Regimens Used during Severe or Acute Hyperglycemia

In this study, the use of sliding-scale insulin regimen was common among the study population. Of 338 cases, 53% involved the use of sliding-scale insulin regimen, despite its use not recommended by the ADA and published journals [5], [7], [8], [9]. The use of sliding-scale insulin regimen is discouraged as it only attempts to treat severe or acute hyperglycemia after it has occurred [10]. According to a published local study, 12% and 83% of admitted patients treated with sliding-scale insulin had at least one episode of hypoglycemia and hyperglycemia, respectively [7].

### Glycemic Control achieved with Insulin Regimens

In this study, the mean insulin dose used in sliding-scale insulin regimens was low (3.14±0.9 units/hour), which is possibly attributable to the insulin titration algorithms used, whereby insulin was administered on hourly basis. On the other hand, a higher mean insulin dose (12.51±5.5 units) was achieved with the basal-bolus insulin regimen. This is primarily because in basal-bolus-treated patients the insulin units were calculated based on the patient's body weight and adjusted appropriately based on the blood glucose levels throughout hospitalization.

The results of this study also demonstrate significant differences in cases of hypoglycemia (defined as blood glucose <3.3 mmol/L) between basal-bolus insulin and sliding-scale insulin regimens (p = 0.005). The use of sliding-scale insulin and basal-bolus insulin resulted in 10.1% and 2.5% cases of hypoglycemia, respectively. This finding may relate to the fact that sliding-scale insulin was used in more admissions than basal-bolus insulin in this study population; the number of blood glucose readings where the sliding-scale insulin regimen was used was double that of basal-bolus insulin.

### Factors Associated with the Management of Severe or Acute Hyperglycemia

#### Causes of Severe or Acute Hyperglycemia

This study demonstrated a significant association between DKA and the use of insulin regimens throughout the severe or acute hyperglycemia phase (p = 0.043), with DKA more common in cases in which sliding-scale insulin was used. Sliding-scale insulin use remained common among the DKA patients, despite recommendations urging its discontinuation [5].

Conversely, cardiovascular disease was also significantly associated with the insulin regimen used (p = 0.005), but the number of cardiovascular disease cases in which basal-bolus insulin was used was approximately double that of sliding-scale insulin. A study focusing on cardiovascular disease reported that the strict control of preprandial and postprandial hyperglycemia resulted in the reduction of cardiovascular disease among T2DM patients [11]. Thus, treatment of severe or acute hyperglycemia secondary to cardiovascular diseases with a basal-bolus insulin regimen is reasonable, where the bolus doses are administered to control the excessive rise of postprandial blood glucose levels.

Six patients were admitted with hyperglycemia secondary to or caused by acute exacerbation of bronchial asthma. All were treated with basal-bolus insulin. The development of severe or acute hyperglycemia following an acute asthma attack could be because of the increase in stress hormones such as cortisol and catecholamines [12]. According to Dungan *et al*. [13], a subcutaneous basal-bolus insulin regimen is a better approach than sliding-scale insulin for achieving effective glycemic control in stress hyperglycemia following an acute illness, which is similar to the findings reported by [8].

### Drug Use during the Severe or Acute Hyperglycemia Phase

The use of certain classes of medications including corticosteroids (p = 0.037), and loop diuretics (p = 0.016) appeared to have a significant influence on the management of severe or acute hyperglycemia.

Corticosteroids are used widely in hospital setting and are known to provoke new-onset hyperglycemia in non-diabetic patients or exacerbate severely uncontrolled hyperglycemia in patients with diabetes [14]. The development of severe or acute hyperglycemia resulting from the administration of corticosteroids occurs primarily because of a decrease in insulin secretion and insulin sensitivity [15]. In this study, the most common corticosteroid dosing regimen encountered was oral prednisolone 30 mg administered once daily.

Furthermore, significant association was observed between the use of loop diuretics and the use of the basal-bolus insulin regimen (p = 0.016). A recent study by Zaman Huri *et al*. [3] revealed that the use of loop diuretics was found to have significant association with insulin resistance in T2DM patients during severe or acute hyperglycemia. The study reported that more patients receiving loop diuretics were insulin resistant (26 patients) compared with those who were insulin sensitive (19 patients). The authors concluded that this may indicate that loop diuretics might increase insulin resistance in T2DM patients during severe or acute hyperglycemia [3].

A limitation of this study relates to its retrospective nature, whereby assessment of glycemic control in the patients studied could be based only on the data available in medical records. A patient's condition throughout the severe or acute hyperglycemia phase could not be assessed, and it was not possible to further investigate decisions on the approach taken by clinicians regarding glycemic control.

## Conclusion

The use of a sliding-scale insulin regimen among T2DM patients with severe or acute hyperglycemia admitted to our institution was common. In addition, we found that DKA, cardiovascular diseases and acute exacerbation of bronchial asthma appeared to have a significant association with the insulin regimens used in glycemic control. Several concurrent medications, including corticosteroids, and loop diuretics were also found to be significantly associated with the insulin regimen used. Overall, this study revealed that lower blood glucose levels were achieved with a basal-bolus insulin regimen compared with sliding-scale insulin in the population studied.

The identification of factors associated with the insulin regimens used in managing severe or acute hyperglycemia may contribute towards achieving optimal glycemic control in T2DM patients. There is currently a lack of published studies on the factors associated with the management of severe or acute hyperglycemia, and further investigation of this is warranted.
